# M379A Mutant Tyrosine Phenol-lyase from *Citrobacter freundii* Has Altered Conformational Dynamics

**DOI:** 10.1002/cbic.202200028

**Published:** 2022-05-24

**Authors:** Robert S. Phillips, Benjamin Jones, Sarah Nash

**Affiliations:** a Department of Chemistry, University of Georgia, Athens, Georgia 30602 (USA); b Department of Biochemistry and Molecular Biology, University of Georgia, Athens, Georgia 30602 (USA); c Department of Biological Engineering, University of Georgia, Athens, Georgia 30602 (USA); d Department of Biology, University of Georgia, Athens, Georgia 30602 (USA)

**Keywords:** amino acids, biocatalysis, kinetics, mutagenesis, protein structures

## Abstract

The M379A mutant of *Citrobacter freundii* tyrosine phenol-lyase (TPL) has been prepared. M379A TPL is a robust catalyst to prepare a number of tyrosines substituted at the 3-position with bulky groups that cannot be made with wild type TPL. The three dimensional structures of M379A TPL complexed with L-methionine and 3-bromo-DL-phenylalanine have been determined by X-ray crystallography. Methionine is bound as a quinonoid complex in a closed active site in 3 of 4 chains of homotetrameric M379A TPL. M379A TPL reacts with L-methionine about 8-fold slower than wild type TPL. The temperature dependence shows that the slower reaction is due to less positive activation entropy. The structure of the M379A TPL complex of 3-bromo-DL-phenylalanine has a quinonoid complex in two subunits, with an open active site conformation. The effects of the M379A mutation on TPL suggest that the mutant enzyme has altered the conformational dynamics of the active site.

## Graphical Abstract

**The M379A mutant** tyrosine phenollyase is a good catalyst for synthesis of L-tyrosine analogues. The crystal structure and kinetic studies show that the mutation affects conformational changes coupled with the catalytic reaction.

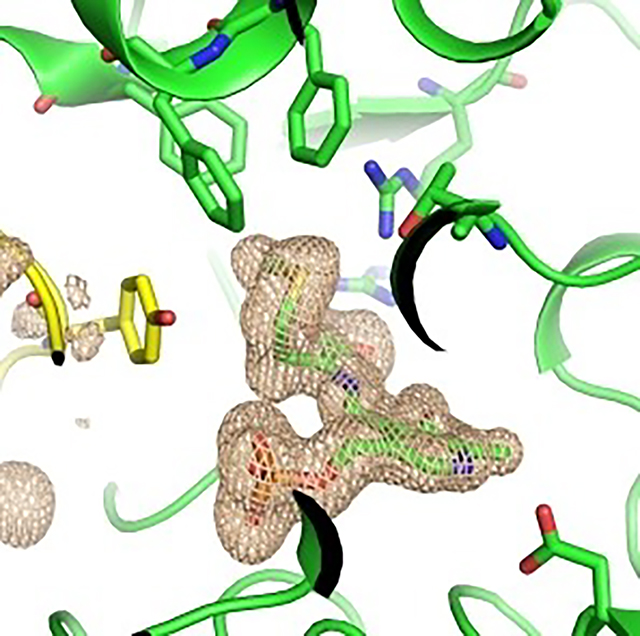

## Introduction

Tyrosine phenol-lyase (TPL, [EC 4.1.99.2]) catalyzes the reversible pyridoxal-5′-phosphate (PLP) dependent β-elimination of L-tyrosine to give phenol and ammonium pyruvate (Equation 1).^[1]^ In addition, TPL catalyzes the irreversible β-elimination of *S*-alkyl and *S*-aryl-L-cysteines (Equations 2 and 3), as well as other amino acids with good leaving groups on the β-carbon, in vitro.^[1–3]^ The reversibility of the TPL reaction in Equation 1 has been used to prepare a number of analogues of L-tyrosine with pharmaceutical and biochemical applications.^[4–7]^ However, the substrate specificity of wild type TPL from *Citrobacter freundii* for phenols is rather narrow, with only phenol and fluorinated phenols giving good yields of amino acid products.^[8]^ Thus, there has been significant interest in mutants of TPL with broader substrate specificity, especially for synthesis of L–DOPA.^[9]^ We have prepared F448A, F448L, and F449A active site mutants of *C. freundii* TPL previously, and we found that all of these show very low (10^−3^ to 10^−4^) elimination activity with L-tyrosine, but retain near wild type activity with *S*-ethyl-L-cysteine (Equation 2) and *S*-(*o*-nitrophenyl)-L-cysteine (Equation 3).^[10,11]^ We have previously obtained X-ray crystal structures of wild-type and F448A mutant TPL with bound substrates and inhibitors.^[11]^ These structures include all of the proposed *gem*-diamine, external aldimine, quinonoid, and aminoacrylate reaction intermediate complexes. The rate of formation of the aminoacrylate intermediate from L-tyrosine shows nonlinear temperature and pressure dependencies, suggesting that the enzyme conformational change from an open to a closed active site is coupled with the C—C bond cleavage step.^[12]^ We have now prepared the M379A mutation of TPL in order to increase the range of substrates for TPL. The results show that M379A has reduced catalytic activity with L-tyrosine, but nevertheless it is a robust catalyst for preparation of 3-substituted tyrosine analogs that cannot be made with wild type TPL. The UV-visible spectra and crystal structures of M379A TPL complexed with L-methionine and 3-bromo-DL-phenylalanine show that quinonoid intermediates form, similar to wild type TPL. However, the results demonstrate that the M379A mutation affects the dynamics of the conformational change coupled with catalysis.

(1)

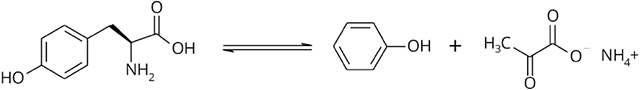



(2)

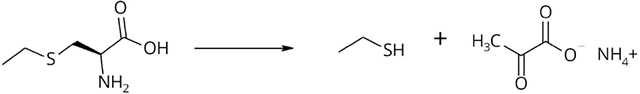



(3)

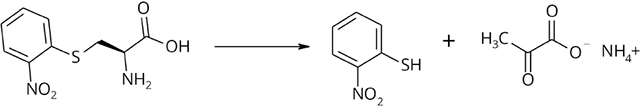



## Results and Discussion

### Catalytic activity of M379A TPL

The catalytic activity of M379A TPL with a range of 3-substituted L-tyrosines was examined, and the results are given in Table 1. These activities were measured with a lactate dehydrogenase (LDH) coupled assay, following the consumption of NADH at 340 nm as pyruvate is produced by TPL.^[13]^ The reaction with L-tyrosine and several other substituted tyrosines was linear with concentration up to the highest concentration studied, so *k*_cat_ could not be determined, but the value of *k*_cat_/*K*_M_ can be determined from the initial slope of the *v* vs [S] plot. The *k*_cat_/*K*_M_ value of L-tyrosine for M379A TPL is reduced more than 100-fold compared with wild-type TPL. However, 3-fluoro and 3-chloro-L-tyrosine show typical Michaelis-Menten kinetics, and much higher activity than L-tyrosine with M379A TPL. For 3-fluoro-L-tyrosine, *k*_cat_ is reduced only 2-fold, and *k*_cat_/*K*_M_ only 20-fold, compared to wild-type TPL, but 3-chloro-L-tyrosine is a very weak substrate for wild type TPL. Larger substituents, bromo, methoxy, and methylthio, showed similar *k*_cat_ values, but lower values of *k*_cat_/*K*_M_, than 3-F—L-tyrosine with M379A TPL (Table 1). This suggests that steric effects are reducing the binding of the substrates more than the catalytic activity. These tyrosines with bulky 3-substituents do not show activity with wild type TPL in the LDH coupled assay. However, 3-bromo and 3-methyl-L-tyrosine have been reported previously to be poor substrates (~2–3%) for wild type TPL using a discontinuous colorimetric reaction for pyruvate.^[5]^ Surprisingly, L–DOPA shows lower activity with M379A than wild type TPL, even though the 3-OH group is smaller than methoxy or methylthio. Thus, the low activity with L–DOPA must be due to the polarity of the 3-OH substituent rather than steric effects. In contrast, the reactions of *S*-ethyl-L-cysteine (Eqn. 2) and *S*-(*o*-nitrophenyl)-L-cysteine (Eqn. 3) have *k*_cat_ and *k*_cat_/*K*_M_ values for M379A TPL comparable to or even greater than those of wild-type TPL (Table 1). Thus, the M379A mutation specifically affects the elimination reaction of tyrosines with TPL.

Synthetic reactions were performed at room temperature in 250 mL reactions, containing 0.4 M ammonium acetate, 0.2 M sodium pyruvate, 0.1 mM PLP, 5 mM 2-mercaptoethanol, and various phenols, at pH 8.2, with 24 mg M379A TPL. The phenol was added in several portions over two days to maintain a concentration below 5 mM, since high concentrations of phenols can cause protein denaturation.^[15]^ The product was purified by cation exchange chromatography on a Dowex 50 column (20×5 cm) in the H^+^ form. The reaction mixture was loaded on the column, and it was washed with water until the pH was neutral. The amino acid was eluted with 2 M NH_3_, and the eluate evaporated in vacuo. The solid residue was crystallized from aqueous ethanol to give the product in 50–90% isolated yield based on phenol as the limiting reagent (Table 2). All phenols tested gave significant yields despite the large differences in the kinetics for the elimination reactions (Table 1). The lowest yield was seen with 2-chlorophenol, possibly because the reaction mixture became cloudy with precipitated protein. This suggests that 2-chlorophenol is a more potent protein denaturant than the other phenols in Table 2. The highest yield was seen with guiacol (2-methoxyphenol).

### The crystal structures of M379A TPL complexed with L-methionine and 3-bromo-DL-phenylalanine

**Figure 1.**
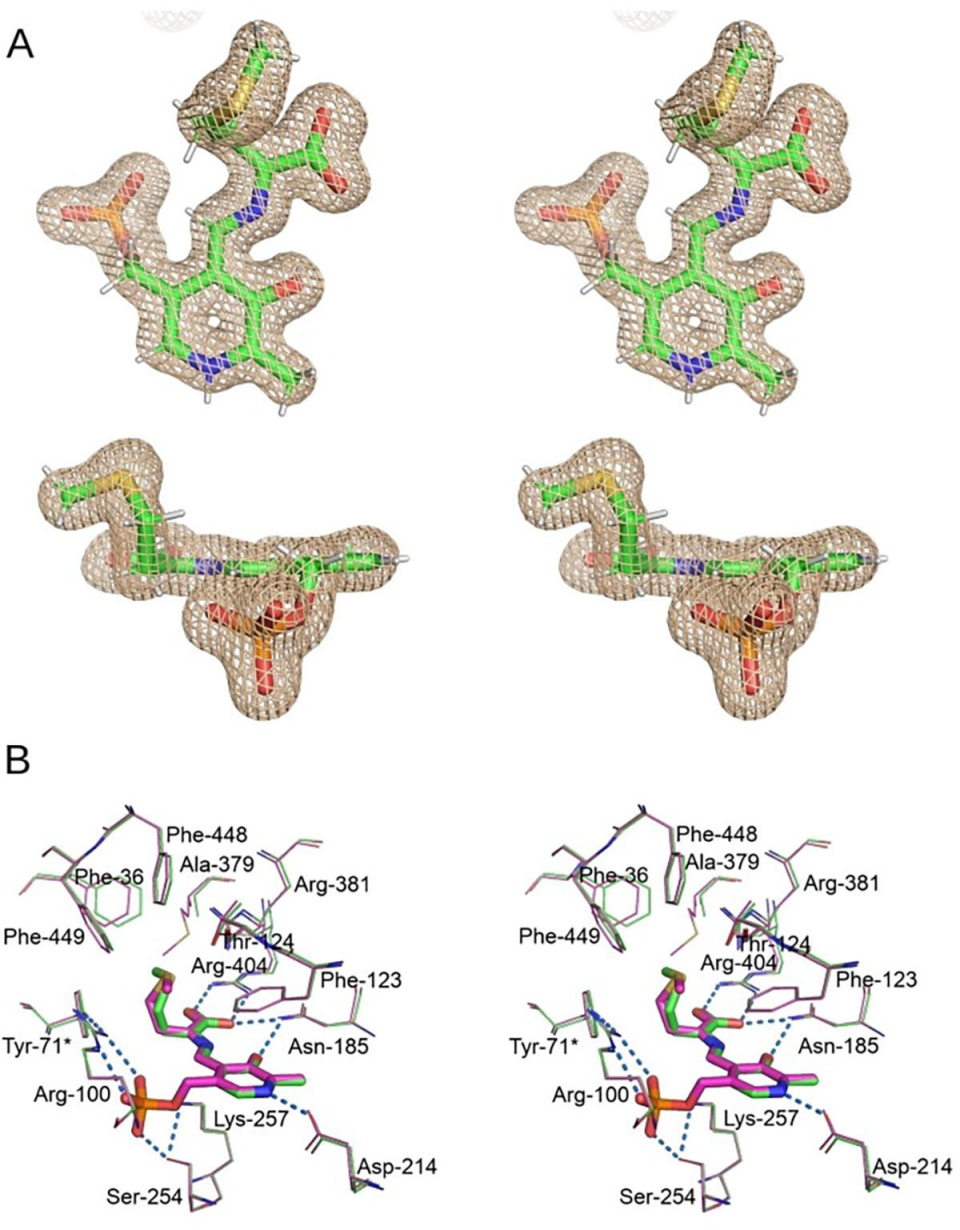
Structure of M379A TPL complexed with L-methionine. A. Crossed-eye stereo view of sim omit mFo-DFc maps at 4 σ of the PLP—L-methionine quinonoid complex in chain A. B. Crossed-eye stereo view of the overlay of the active site structure of M379A (green) and wild type TPL (magenta) complexed with L-methionine. Hydrogen bonds are indicated with blue dashes.

M379A TPL was crystallized by a new method, and the structure of the complex with L-methionine was determined by X-ray crystallography. The complexes were prepared by soaking the crystals in a cryosolvent solution containing the ligand before flash cooling in liquid nitrogen and data collection. The structure was determined to a resolution of 1.37 Å and refined to a final R_free_ of 15.5%. This structure has been deposited in the Protein Databank with accession code **7TCS**, and the data collection and refinement statistics are given in Table 3. In contrast to previous structures of wild type TPL, which has a homodimer in the asymmetric unit in the *P*2_1_2_1_2 space group, the asymmetric unit is the homotetramer of M379A TPL, in the P1 space group, and electron density of bound L-methionine complexed with PLP is clearly seen in three of four subunits (Figure 1A). These subunits are all found in a closed conformation of the active site, with the L-methionine bound in a quinonoid complex, as was previously found with the L-methionine complex of wild-type TPL.^[16]^ The structures of chain A of the L-methionine complexes of wild type and M379A TPL align with an overall RMSD of 0.28 Å (Figure 1B). However, the conformation of the bound L-methionine side chain is different in M379A TPL than wild-type TPL (Figure 1B), since the extra space in the active site allows the methyl group to occupy an *anti* rather than a *syn* orientation with respect to the PLP ring. Thus, the M379A mutation has increased the volume of the substrate binding site, without affecting the positions of the other active site residues, except for Phe-36, which moves about 1 Å to partially fill the space created by the mutation (Figure 1B). The S of Met-379 moves about 1.7 Å toward the side chain of the bound L-methionine in the closed conformation of wild type TPL, filling in the space in the active site. There are no new structured water molecules bound in the cavity created by the M379A mutation. The hydrogen bonding distances between the PLP N1 of the methionine quinonoid complex and OD2 of Asp-214 (2.79 Å), and NH1 (2.75 Å) and NH2 (2.72 Å) of Arg-404 with the methionine carboxylate, are identical for M379A and wild type TPL.

Another difference between the structures of M379A and wild-type TPL complexed with L-methionine is seen in chain D. In the wild-type TPL structure with L-methionine, chain B has no bound ligand and has an open conformation of the active site, while in the M379A TPL structure, chain D has no bound ligand, but is present as a mixture of open and closed conformations, in an approximate 60:40 ratio (Figure S1). We found previously that the complex of F448A mutant TPL with L-methionine is in a 52:48 mixture of open and closed conformations;^[11]^ however, this is the first observation of a mixture of open and closed conformations in an uncomplexed subunit of TPL. This suggests that the M379A mutation affects the active site equilibrium of TPL between open and closed conformations.

Crystals of M379A TPL were also soaked with 3-bromo-DL-phenylalanine to evaluate how bulky substituents at the 3-position of tyrosine affect substrate binding. The structures of wild-type and F448A TPL complexed with L-phenylalanine have been determined previously.^[11]^ Those structures were found to contain external aldimines in open conformations in chain B, and quinonoid complexes in chain A, with a mixture of open and closed conformations for wild type TPL, but predominantly an open conformation for F448A TPL. The structure of M379A TPL with 3-bromophenylalanine bound has been submitted to the Protein Databank with accession code **7TDL**, and has a resolution of 1.6 A with R_free_ of 22%. The structure shows the ligand bound to chain A, and partially bound to chain B.

**Figure 2.**
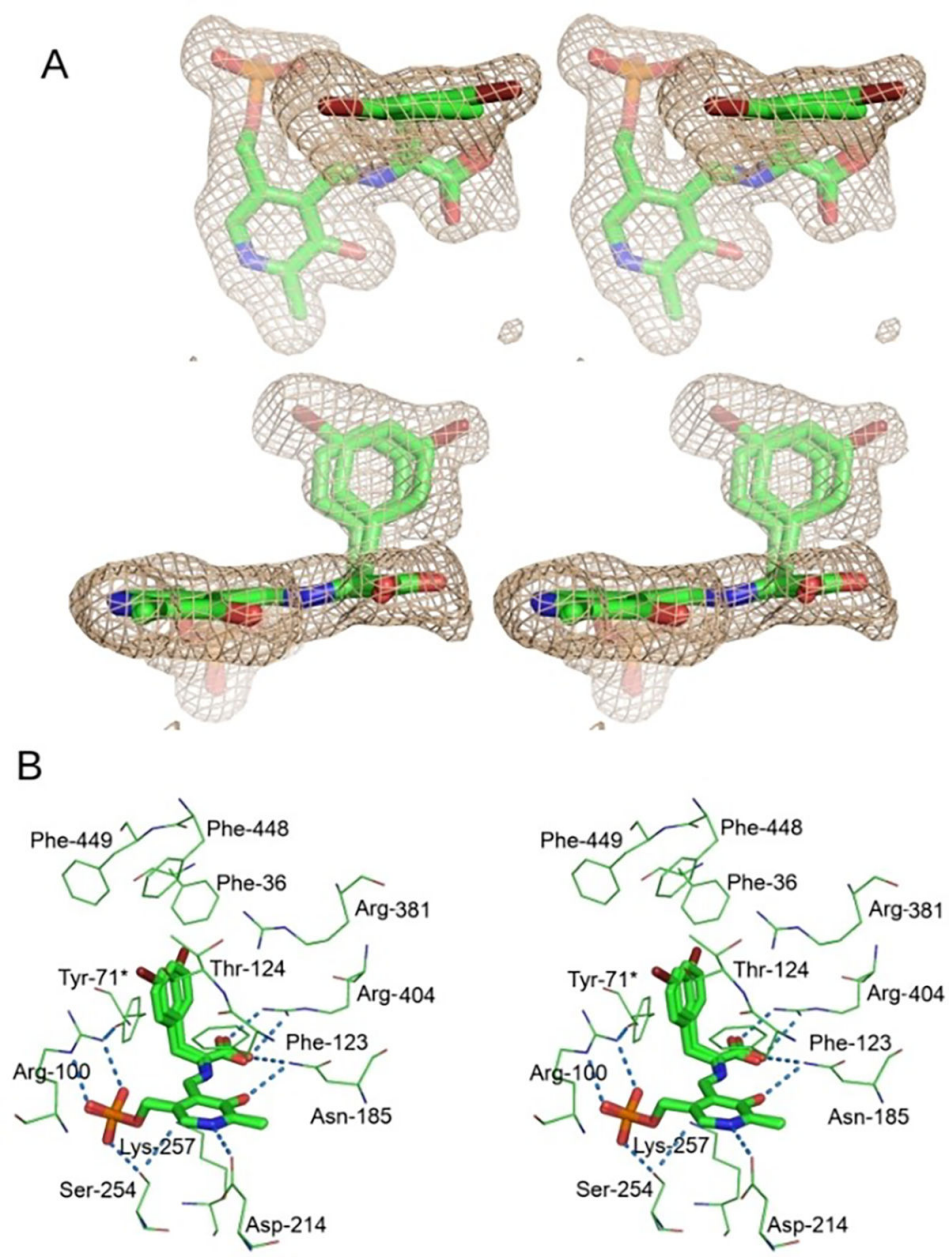
Structure of M379A TPL complexed with 3-bromo-DL-phenylalanine. A. Crossed-eye stereo view of sim omit mFo-DFc maps at 4 σ of the PLP—L-3-bromophenylalanine quinonoid complex in chain A. B. Crossed-eye stereo view of the overlay of the active site structure of M379A chain A complexed with 3-bromo-DL-phenylalanine. Hydrogen bonds are indicated with blue dashes.

3-Bromo-DL-phenylalanine is bound to the PLP in chain A as a quinonoid complex since the electron density is coplanar at the α-carbon with the PLP ring (Figure 2A). The electron density requires fitting the ring of the 3-bromophenylalanine as two rotamers, with the bromine oriented either *syn* or *anti* to the PLP ring, in 80:20 ratio, respectively. There is no remaining electron density between the NZ of Lys-257 and C4’ of the PLP, indicating full occupancy of the ligand. Surprisingly, in contrast to the structure with L-methionine, chain A is in an open conformation, as can be seen from the positions of Phe-448 and Phe-449 (compare Figure 1B and 2B). In chain B, there is partial occupancy of 3-bromophenylalanine as a quinonoid complex, with the remainder of the PLP bound to Lys-257, and the active site is also in an open conformation (Figure S1). Although chains C and D contain no bound amino acid ligand, they are present as fully closed conformations, as seen by the positions of Phe-448 and Phe-449, with a chloride ion occupying the carboxylate binding site, between Arg-381 and Arg-404 (Figure S2). The previous structures of TPL did not show a closed conformation of the internal aldimine, and did not have a chloride bound in the carboxylate binding site. It is possible that the chloride binding is due to the increased active site volume arising from the M379A mutation.

### Reaction of M379A TPL with L-methionine

**Figure 3.**
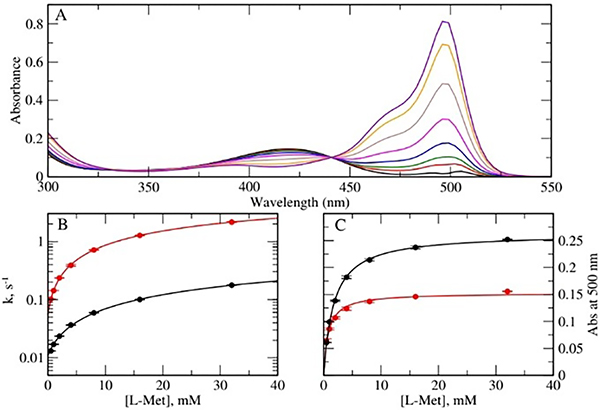
Reaction of M379A TPL with L-methionine. A. Reaction of M379A TPL (19 μM) in 0.05 M potassium phosphate, pH 8.0, with 20 mM L-methionine. Scans are shown at 0.096 s (black), 1 s (red), 2 s (green), 4 s (blue), 8 s (magenta), 16 s (brown), 32 s (orange) and 58 s (indigo). B. Dependence of *k*_obs_ on [L–Met]. Black, M379A TPL; red, wild type TPL. The lines are the curves from fitting the data to Equation 5. C. Dependence of the absorbance change at 500 nm on [L–Met]. Black, M379A TPL; red, wild type TPL. The lines are the curves from fitting the data to equation 6.

**Figure 4.**
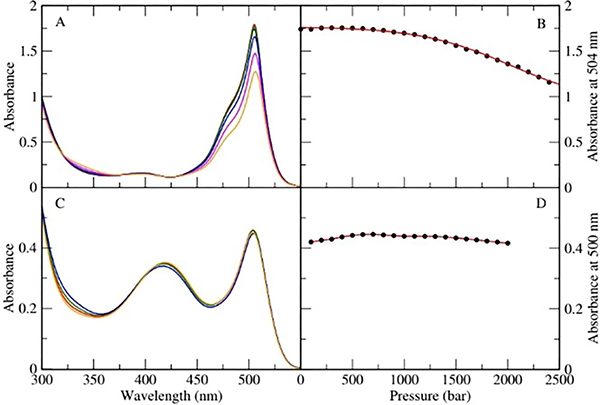
Effect of hydrostatic pressure on the absorbance spectra of M379A and wild-type TPL complexed with L-methionine. A. Effect of pressure on the M379A TPL complex with L-methionine. B. Absorbance at 504 nm as a function of hydrostatic pressure. The line is the calculated curve from fitting to Eqn. 8 with the parameters given. C. Effect of hydrostatic pressure on the wild-type TPL complex with L-methionine. D. Absorbance at 500 nm as a function of hydrostatic pressure. The line is from connecting the dots.

L-Methionine is a substrate analog for TPL, which has been useful to probe the mechanism of substrate binding.^[11,16–18]^ Mixing of M379A TPL with L-methionine results in formation of a strong absorbance peak at 500 nm, assigned to a quinonoid intermediate, similar to what was seen before with wild type TPL (Figure 3A). The intense absorbance of the quinonoid peak at 500 nm is consistent with the crystal structure showing 3 of 4 chains of the tetramer containing quinonoid complexes. These kinetic data fit very well to a single exponential process. The observed rate constant, *k*_obs_, for the reaction is a function of [L-methionine], as shown in Figure 4B. These concentration dependence data show a hyperbolic dependence, consistent with the mechanism in Equation 4, with a rapid initial binding equilibrium to form an external aldimine complex, followed by a slower reaction to form the 500 nm quinonoid peak, where *k*_f_ is the rate constant in the forward direction for quinonoid intermediate formation, *k*_r_ is the rate constant in the reverse direction, and *K*_d_ is the dissociation constant for formation of the external aldimine of L-methionine (Equation 5). M379A TPL has values of *k*_f_=0.976±0.133 s^−1^, *k*_r_=0.011±0.0007 s^−1^, and *K*_d_=157±25.5 mM from fitting the data in Figure 3B to Equation 5. Wild-type TPL bound with L-methionine shows a similar spectrum, but with a lower intensity of the 500 nm absorbance peak. However, wild type TPL has values of *k*_f_ = 7.67±0.25 s^−1^, *k*_r_=0.055±0.004 s^−1^, and *K*_d_=85.5±3.7 mM for the reaction of L-methionine. These values for wild type TPL are very similar to those obtained previously.^[19]^ Thus, the mutation has only a 2-fold effect on the equilibrium constant for formation of the external aldimine, but causes an 8-fold decrease in *k*_f_ and a 5-fold decrease in *k*_r_ for quinonoid intermediate formation. The total amplitude of the absorbance changes at 500 nm are also concentration dependent (Figure 3C). Fitting the data in Figure 3C to a hyperbolic binding isotherm in Equation 6 gives overall *K*_d_ values for L-methionine binding of 1.74±0.05 mM for M379A TPL and 0.79±0.07 mM for wild type TPL. Thus, despite the differences in kinetics of quinonoid intermediate formation, the overall binding equilibrium of L-methionine is only affected 2-fold by the M379A mutation.


(4) $$\text{E}\;+\;\text{Met}\;\overset{{\text{K}}_{d}}{⇌}{\text{E-Met}}_{\text{AL}}\overset{k_{f}}{\underset{k_{r}}{⇌}}{\text{E-Met}}_{\text{Q}}$$

(5) $$k_{\text{obs}}=k_{\text{f}}{}^{*}[\text{Met}]/\left(K_{\text{d}}+[\text{Met}]\right)+k_{\text{r}}$$

(6) $$\text{A}={\text{A}}_{\text{max}}{}^{*}[\text{Met}\;]/\left(K_{\text{d}}+[\text{Met}\;]\right)$$

The effect of temperature on the kinetics of quinonoid intermediate formation with L-methionine for wild type and M379A TPL was investigated in the range from 8 to 48°C, with the results shown in Figure S3. These data were fit to Equation 7, the macromolecular rate equation,^[20]^ where k_B_ is the Boltzmann constant, *h* is Planck’s constant, T is the temperature in Kelvin, T_o_ is the reference temperature, 298.1 K, ΔH_To_^ᙾ^ is the activation enthalpy at T_o_, Δs_To_^ᙾ^ is the activation entropy at T_o_, and ΔC_p_^ᙾ^ is the activation heat capacity. Including a transition state heat capacity change, ΔC_p_^ᙾ^, gives a slightly better fit than the Eyring equation. However, the resulting values of ΔH^ᙾ^ and ΔS^ᙾ^ are very similar using either equation for fitting. The results of fitting to Equation 7 are given in Table 4.


(7) $$k={\left({\text{k}}_{B}\text{T}/h\right)}^{*}\exp(\left({{\text{-ΔH}}_{\text{To}}}^{ᙾ}{{\text{-ΔC}}_{\text{p}}}^{ᙾ*}\left({\text{T-T}}_{o}\right)\right)/\text{RT}\;+\left({{\text{ΔS}}_{\text{To}}}^{ᙾ}+{{\text{ΔC}}_{\text{p}}}^{ᙾ}\left(\ln\text{T-ln}{\text{T}}_{o}\right)\right)/\text{R})$$

There is no difference in the values of ΔH^ᙾ^ for the M379A mutant and wild type enzyme, but there is a significant difference in the values of ΔS^ᙾ^, with a ΔΔS^ᙾ^ of 19 J/mol-K. This corresponds to a ΔΔG^ᙾ^ of −5.7 kJ/mol at 298 K for the reaction of wild type TPL, resulting in a 10-fold increase in the reaction rate. The ratio of *k*_f_ for wild-type and M379A TPL is 7.67/0.967=7.93. Thus, the slower reaction kinetics of M379A TPL to form a quinonoid complex with L-methionine can be entirely explained by the activation entropy difference. There are also significant differences in the heat capacities, which are negative for wild type and positive for M379A TPL. This is consistent with altered vibrational coupling of the transition state^[21]^ for quinonoid intermediate formation by the mutant TPL.

The effect of hydrostatic pressure on the spectrum of the M379A-TPL L-methionine complexes was also examined (Figure 4). The spectrum of the complex shows a significant decrease in absorbance of the 504 nm peak at pressures above 1 kbar (Figure 4A and 4B). The absorbance changes are reversible on decompression, so the changes are not due to irreversible transamination, which occurs slowly with the L-methionine complex of wild-type TPL. The decrease in absorbance can be fit to a Boltzmann function (Equation 8), where A_p_ is the absorbance at pressure P, ΔA is the absorbance difference, *K*_o_ is the pressure-independent value of the equilibrium constant, and ΔV is the reaction volume. The fit gives an apparent *K*_d_ of 98.7±19.2 and a ΔV of +56.2±4.0 mL/mol for quinonoid intermediate formation. This is in good agreement with the equilibrium constant of *k*_f_/*k*_r_=88.7 for quinonoid complex formation calculated from the kinetic data in Figure 3B. These results are consistent with a pressure dependent shift of the equilibrium toward an open conformation, which has a smaller system volume due to the solvation of exposed hydrophobic protein surface. We showed previously that the number of bound water molecules in a protein conformational equilibrium can be estimated from the approximate 20% apparent volume increase between protein bound and free water molecules.^[22]^ In the case of M379A TPL, the volume change corresponds to about 16 waters required to solvate the increased protein surface in the open conformation. In contrast, there is very little change in the spectrum of the wild type TPL L-methionine complex at pressures up to 2 kbar (Figures 4C and 4D). These changes are too small to fit reliably to a Boltzmann function. This suggests that the conformational equilibrium position lies more toward the closed conformation for wild-type TPL. Consistent with this interpretation, the equilibrium constant for quinonoid complex formation can be calculated from the *k*_f_/*k*_r_=139 in Figure 3B.


(8) $$A_{p}=\Delta A*\left({K_{o}}^{*}\text{exp}(-\text{PΔV}/\text{RT})\right)/\left(1+{K_{o}}^{*}\text{exp}(-\text{PΔV}/\text{RT})\right)+A_{o}$$

### Reaction of M379A TPL with 3-bromo-DL-phenylalanine

**Figure 5.**
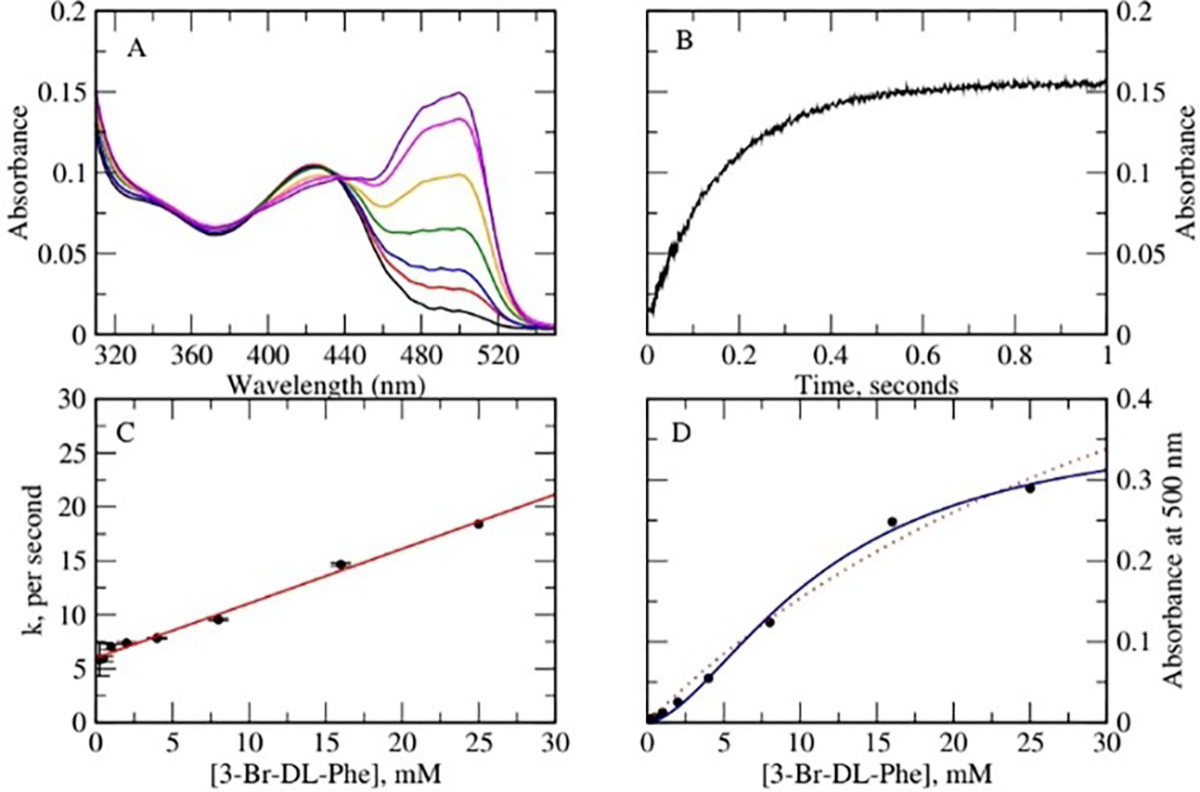
Reaction of M379A TPL with 3-bromo-DL-phenylalanine. A. Spectra of M379A TPL during reaction with 10 mM 3-bromophenylalanine. Black, 0.010 s; red, 0.020 s; blue, 0.040 s; green, 0.080 s; orange, 0.160 s; magenta, 0.320 s; violet, 0.640 s. B. Time course of the reaction at 500 nm. C. Dependence of *k*_obs_ on [3-Br-DL-Phe]. D. Dependence of the absorbance change at 500 nm on [3-Br-DL-Phe]. Dashed line, fit to a hyperbolic binding equation. Solid line, fit to a cooperative binding equation.

Mixing of M379A TPL with 50 mM 3-bromo-DL-phenylalanine results in rapid formation of a broad quinonoid absorption peak at 500 nm, shown in Figure 5A. The absorbance intensity of the peak is much less than that of the L-methionine complex. The time course data fit well to a single exponential process, significantly faster than the reaction of M379A TPL with L-methionine (Figure 5B). The concentration dependence of *k*_obs_ on [3-Br-DL-phenylalanine] is linear up to 25 mM (Figure 5C), so it can be fit to a simple first order polynomial equation, with an apparent second order rate constant of (5.1±0.2) × 10^2^ M^−1^ s^−1^ and a *k*_r_ of 6.0±0.2 s^−1^. This indicates that the compound has a *K*_d_ > 25 mM for formation of the external aldimine intermediate. Thus, based on Equation 5, *k*_obs_=*k*_f_/*K*_d_ at concentrations ⪡*K*_d_. Unfortunately, higher concentrations of 3-bromo-DL-Phe are limited by the low solubility of the amino acid. The absorbance of the peak also increases with concentration. Surprisingly, the absorbance data fit better to a binding equation with cooperativity (Figure 5D, solid line), with *K*_d_=11.5±1.8 mM and n=1.67±0.20, rather than Equation 6 (dashed line). However, there is no previous evidence for cooperativity in ligand binding or catalysis by TPL. In addition, the width of the 500 nm peak in Figure 5A is also much broader than that of the L-methionine quinonoid complex (Figure 4A). This may be the result of the two different rotamers of the bromophenyl ring seen in the crystal structure (Figure 2), if each rotamer has a slightly different absorption spectrum. Regardless, the single exponential kinetics demonstrates that the rotamers do not have significantly different rates of formation.

## Conclusion

Wild type TPL has a rather narrow substrate specificity for L-tyrosine, with fluorinated tyrosines the only analogues showing good activity.^[23]^ Methionine-379 is located on the small domain in the active site of TPL, where it is within van der Waals contact distance with the bound substrate. Thus, it is a likely target for mutagenesis to expand the size of the active site, in order to increase the range of substrates. TPL has been used extensively for the synthesis of L-tyrosine and analogues.^[23–28]^ The enzyme shows very high stereoselectivity for L-tyrosine, with no detectable D-amino acid formed.^[26,28]^ Previously, M379V TPL was identified as a suitable catalyst for synthesis of L-tyrosines with bulky substituents that were not readily synthesized by wild type TPL.^[6,7, 25–28]^ We have now prepared M379A TPL, and we find that it is also a robust catalyst to prepare 3-substituted L-tyrosines, with the notable exception of L–DOPA. However, the isolated yield from the reaction of guiacol (2-methoxyphenol) is excellent (Table 2), and 3-methoxytyrosine is easily converted to L–DOPA simply by reflux in concentrated HBr or HI.^[29]^ Thus, the lack of reactivity of L–DOPA is not due to steric effects, but may be the result of unfavorable binding of a polar substrate in a hydrophobic pocket. The product yields in Table 2 are similar to those reported for M379V TPL.^[6,7,25–28]^

The crystal structures of M379A with L-methionine and 3-bromophenylalanine bound were obtained. These structures were refined to resolutions of 1.37 and 1.6 Å, respectively, considerably higher resolution than previous structures of wild type or F448A mutant TPL. The structure of the L-methionine complex shows that a quinonoid complex with PLP is formed, similar to wild type TPL, with a closed active site conformation. This structure superposes very closely on the wild type TPL structure, indicating that the mutation has no overall effect on the protein structure. In contrast, the structure with 3-bromophenylalanine has the bound amino acid in a quinonoid complex, but the active site is open. This may be the result of steric effects of the bromine on Phe-448 and Phe-449, which come into the active site in the closed conformation and make van der Waals contacts with the substrate.^[16,30]^ The complex of wild type TPL with L-phenylalanine was previously found to be a mixture of quinonoid and aldimine complexes in open and closed conformations. Furthermore, the 3-bromophenyl ring is in two rotamers, with the bromine orientated *syn* or *anti* with the PLP ring, with the major form being the syn conformation. Previously, the complexes of 3-fluoro-L-tyrosine with Y71F and H448H TPL were found to be exclusively in the *anti* rotamer.^[30]^ Thus, it is likely that the *anti* rotamer is the catalytically active conformation. The reduction in *k*_cat_/*K*_M_ values for tyrosines with larger substituents (Table 1) may be due to the preference for the unproductive *syn* rotamer, combined with steric effects on the formation of the catalytically active closed conformation.

**Scheme 1.**
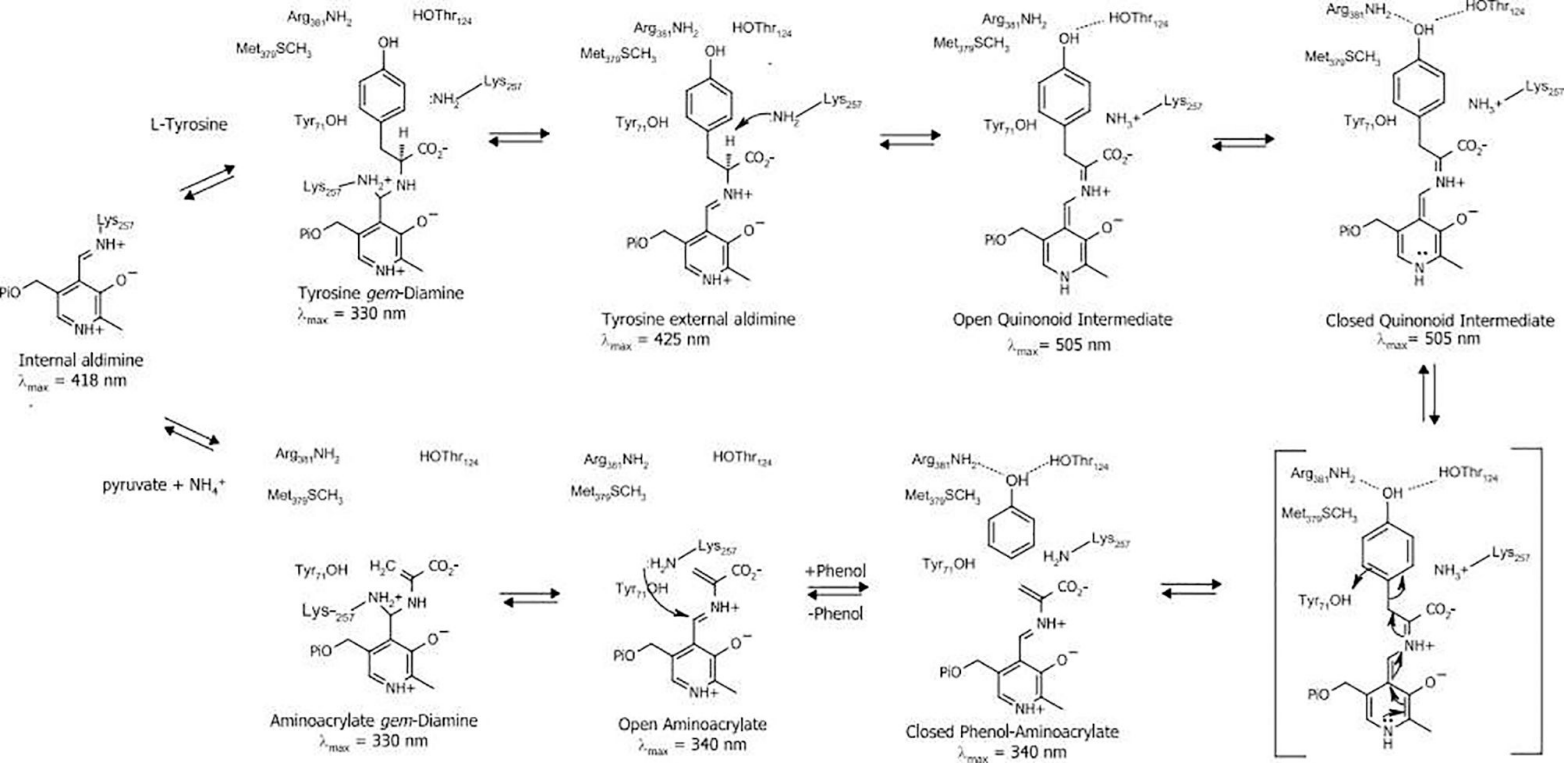
Mechanism of TPL.

The reaction mechanism of TPL is shown in Scheme 1. Initial binding of L-tyrosine to the PLP proceeds through a *gem*-diamine to form the external aldimine. Deprotonation of the α-carbon of the external aldimine results in a quinonoid complex in an open conformation. Closure of the active site then results in a strained complex with hydrogen bonds between the substrate OH with Arg-381 and Thr-124.^[30]^ Elimination occurs with cleavage of the C_β_-Cγ bond and proton transfer to the Cγ from Tyr-71,^[3]^ resulting in a closed aminoacrylate complex with phenol. Subsequent opening of the active site releases phenol, followed by ammonium pyruvate.

Both wild type and M379A TPL form quinonoid complexes with L-methionine with a peak at about 500 nm and a closed conformation of the active site. However, the kinetics of quinonoid intermediate formation are about 8-fold slower for M379A. The temperature dependence of the kinetics show that the slower rate constant is exclusively due to a decrease in the activation entropy for M379A TPL. This is consistent with quinonoid intermediate formation requiring the closure of the active site. Closing of the active site results in release of water molecules bound to polar groups and solvating hydrophobic surfaces, increasing entropy. The effects of hydrostatic pressure on the quinonoid intermediate of M379A TPL with L-methionine, but not wild type, are consistent with this hypothesis, since there is a larger volume for the quinonoid complex than the external aldimine complex, due to release of bound water. Thus, the mutation of Met-379 to alanine has affected the reaction conformational dynamics for TPL. We have previously shown from the effects of temperature and pressure on aminoacrylate intermediate formation in tryptophan synthase that the conformational equilibrium between open and closed conformations is coupled with the catalytic mechanism.^[12]^ The effects of mutation on the conformational equilibrium should be considered in the design of TPL variants with altered substrate specificity. Nevertheless, despite these detrimental effects of the mutation, M379A TPL is a robust catalyst with a broader substrate specificity for synthesis of 3-substituted L-tyrosine analogues.

## Experimental Section

### Synthesis of 3-methoxy-L-tyrosine:

Ammonium acetate (4.25 g, 55 mmol) and sodium pyruvate (2.75 g, 25 mmol) were dissolved in 250 mL water, and the pH was adjusted to 8.3 with 30% aqueous NH_3_, then 3.4 mg PLP, 87.5 μL of 2-mercaptoethanol, and 140 μL of guiacol (1.25 mmol) were added with stirring, followed by 200 μL (24 mg) M379A TPL. After 3 hours at ambient temperature, TLC (RP-18, 10% MeOH) showed a strong UV and ninhydrin positive spot, so another 140 μL of guiacol was added with stirring. The reaction mixture was left stirring at ambient temperature overnight. Three more 140 μL portions of guiacol were added the next day, with several hours between each addition, for a total of 700 μL (6.25 mmol). The following day, the reaction mixture was loaded on a 5×20 cm column of Dowex 50 (H^+^). The column was washed with water until neutral, and the amino acid was eluted with 200 mL of 2 M NH_3_. The collected fraction was evaporated in vacuo, resuspended in 10 mL 50% EtOH, and filtered to give 1.2 g (5.7 mmol, 90% based on limiting guiacol) of 3-methoxy-L-tyrosine. ^1^H-NMR (400 MHz, D_2_O): δ 6.83 (s, 1H), 6.79 (d, J=8.0 Hz, 1H), 6.69 (d, J=7.8 Hz, 1H) 4.22 (m, 1H), 3.74 (s, 3H), 3.18 (dd, J=14.6, 3.0 Hz, 1H), 3.05 (dd, J=14.4, 7.4 Hz, 1H).

### Crystallization of M379A TPL:

M379A TPL was purified as described previously.^[14]^ The purified enzyme was exchanged into 0.1 M HEPES—K, pH 7.5, 0.2 mM PLP, 1 mM DTT, 1 mM EDTA at 10 mg/mL. Two μL of M379A TPL (12 mg/mL in 0.1 M HEPES—K, pH 7.5, 0.1 mM PLP, 1 mM EDTA and 1 mM DTT) was mixed with two μL of 0.1 M HEPES—K, pH 7.5, 0.1 mM PLP, 1 mM EDTA, 1 mM DTT, 19% PEG 5000 MME and 0.3–0.4 M CsCl. The crystals were transferred to a cryosolvent containing 0.1 M HEPES—K, pH 7.5, 0.1 mM PLP, 1 mM DTT, 1 mM EDTA with 25% PEG 5000 MME, 0.2 M KCl, 10% ethylene glycol : glycerol : DMSO (1:1:1), and either 0.1 M L-methionine or 50 mM bromo-DL-phenylalanine sodium salt, incubated for 1–2 minutes, and flash cooled in liquid nitrogen.

### Structure determination:

Data were collected at the SER-CAT ID-22 beamline at 100 K with 0.25° oscillation for 360°. The data were integrated with AutoPROC^[31]^ using XDS.^[32]^ The resolution limits were determined based on CC(1/2)=0.3.^[33]^ The data were phased with PHASER^[34]^ using 2VLH, the L-methionine complex of wild type TPL, as the search model. The resulting model was built with COOT^[35]^ and refined with PHENIX.refine.^[36]^

## Supplementary Material

Supporting information

## Figures and Tables

**Table 1.** Catalytic activity of M379A TPL.

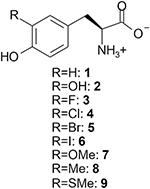				
	M379A TPL		Wild Type TPL	
Substrate	*k*_cat_, s^−1^	*k*_cat_/*K*_M_, M^−1^ s^−1^	*k*_cat_, s^−1^	*k*_cat_/*K*_M_, M^−1^ s^−1^

L-tyrosine (**1**)	ND^[a]^	148 ± 4^[b]^	3.5^[c]^	1.75 × 10^4[c]^
L-DOPA (**2**)	ND^[a]^	0.67 ± 0.15^[b]^	ND^[a]^	27.2 ± 0.1^[b]^
3-F–L-tyrosine (**3**)	0.73 ± 0.01^[b]^	774 ± 2^[b]^	1.4^[c]^	1.4 × 10^4[c]^
3-Cl–L-tyrosine (**4**)	0.38 ± 0.05^[b]^	430 ± 130^[b]^	ND^[a]^	< 4
3-Br–L-tyrosine (**5**)	0.25 ± 0.04^[b]^	60 ± 5^[b]^	NA^[d]^	NA^[d]^
3-I–L-tyrosine (**6**)	NA^[d]^	NA^[d]^	NA^[d]^	NA^[d]^
3-MeO–L-tyrosine (**7**)	0.81 ± 0.12^[b]^	91 ± 9^[b]^	NA^[d]^	NA^[d]^
3-Me–L-tyrosine (**8**)	0.10 ± 0.02^[b]^	9.7 ± 1^[b]^	NA^[d]^	NA^[d]^
3-MeS- L-tyrosine (**9**)	ND^[a]^	44.2 ± 7.2^[b]^	NA^[d]^	NA^[d]^
*S*-ethyl-L-cysteine	ND^[a]^	210 ± 1.9^[b]^	3.9^[c]^	591^[c]^
*S*-(*o*-nitrophenyl)-L-cysteine	7.1 ± 0.1	(9.6 ± 0.1) × 10^4[b]^	5.1^[c]^	4.6 × 10^4[c]^

[a] ND, not determined.

[b] Standard error.

[c] From reference [14].

[d] NA, no activity detected.

**Table 2.** Synthesis of 3-substituted L-tyrosines with M379ATPL.

Phenol substituent	Yield (g)	Yield (%)

2-F	0.99	67
2-Cl	0.80	49
2-Me	1.13	86
2-MeO	1.20	90
2-MeS	1.19	74

**Table 3.** Data collection and refinement statistics.^[a]^

	7TCS	7TDL
	M379A TPL + L–Met	M379ATPL + 3-Br-DL-Phe

Wavelength	1.0000 Å	1.0000 Å
Resolution range	45.27–1.37 (1.419–1.37)	58.41–1.60 (1.657–1.60)
Space group	*P*1	*P*1
Unit cell	63.55 82.99 96.58 93.734 105.714 114.236	61.25 82.87 94.33
Total reflections	2947457 (147792)	113.163 96.537 102.208 1618343 (109728)
Unique reflections	335308 (27495)	213258 (21321)
Multiplicity	8.8 (5.4)	7.6 (5.1)
Completeness (%)	93.98 (77.09)	98.65 (94.49)
Mean I/sigma(I)	12.74 (1.38)	6.48 (0.46)
Wilson B-factor	20.01	24.92
R-meas	0.09114 (1.323)	0.1522 (2.799)
CC1/2	0.998 (0.274)	0.997 (0.333)
CC*	0.999 (0.583)	0.999 (0.707)
Reflections used in refinement	335276 (27491)	211763 (20350)
Reflections used for R-free	2001 (177)	1985 (190)
R-work	0.1412 (0.2991)	0.1817 (0.3598)
R-free	0.1550 (0.3635)	0.2202 (0.3693)
CC(work)	0.978 (0.749)	0.975 (0.647)
CC(free)	0.982 (0.569)	0.969 (0.599)
Number of non-hydrogen atoms	17662	16355
macromolecules	15873	14734
ligands	197	219
solvent	1682	1486
Protein residues	1824	1824
RMS(bonds)	0.116	0.008
RMS(angles)	2.14	1.07
Ramachandran favored (%)	98.01	97.95
Ramachandran allowed (%)	1.88	2.05
Ramachandran outliers (%)	0.11	0.00
Rotamer outliers (%)	0.60	0.84
Clashscore	2.10	3.59
Average B-factor	28.10	35.73
macromolecules	27.08	35.50
ligands	38.63	39.16
solvent	37.02	37.72
Number of TLS groups	17	16

[a] Statistics for the highest-resolution shell are shown in parentheses.

**Table 4.** Activation parameters for reaction of M379A and wild type TPL with L-methionine.

Enzyme	ΔH^ᙾ^, kJ/mol	ΔS^ᙾ^, J/mol K	ΔC_p_^ᙾ^, J/mol K

M379A TPL	105.6 ± 0.6^[a]^	93.9 ± 2.2^[a]^	456 ± 96^[a]^
Wild type TPL	105.6 ± 1.4^[a]^	113 ± 4.9^[a]^	−501 ± 241^[a]^

[a] Standard error.

## Data Availability

The data that support the findings of this study are available from the corresponding author upon reasonable request.

## References

[1] Kumagai H, Yamada H, Matsui H, Ohkishi H, Ogata K, J. Biol. Chem 1970, 245, 1767–1772. 4908868

[2] Phillips RS, Arch. Biochem. Biophys 1987, 256, 302–310. 3111376 10.1016/0003-9861(87)90450-4

[3] Chen HY, Demidkina TV, Phillips RS, Biochemistry 1995, 34, 12276–12283. 7547970 10.1021/bi00038a023

[4] Enei H, Matsui H, Okumura S, Yamada H, Biochem. Biophys. Res. Commun 1971, 43, 1345–1349. 4998713 10.1016/s0006-291x(71)80021-9

[5] Nagasawa T, Utagawa T, Goto J, Kim CJ, Tani Y, Kumagai H, Yamada H, Eur. J. Biochem 1981, 117, 33–40. 7262088 10.1111/j.1432-1033.1981.tb06299.x

[6] Seisser B, Zinkl R, Gruber K, Kaufmann F, Hafner A, Kroutil W, Adv. Synth. Catal 2010, 352, 731–736.

[7] Munozvilla JH, Loo CE, Schwans JP, FASEB J 2020, 34, 1–1.

[8] Phillips RS, Von Tersch RL, Secundo F, Eur. J. Biochem 1997, 244, 658–663. 9119037 10.1111/j.1432-1033.1997.00658.x

[9] Koyanagi T, Katayama T, Suzuki H, Nakazawa H, Yokozeki K, Kumagai H, J. Biotechnol 2005, 115, 303–306. 15639092 10.1016/j.jbiotec.2004.08.016

[10] Phillips RS, Vita A, Spivey JB, Rudloff AP, Driscoll MD, Hay S, ACS Catal. 2016, 6, 6770–6779.

[11] Phillips RS, Craig S, Biochemistry 2018, 57, 6166–6179. 30260636 10.1021/acs.biochem.8b00724

[12] Phillips RS, Craig S, Kovalevsky A, Gerlits O, Weiss K, Iorgu AI, Heyes DJ, Hay S, ACS Catal. 2019, 10, 1692–1703.

[13] Kiick DM, Phillips RS, Biochemistry 1988, 27, 7333–7338. 3207679 10.1021/bi00419a023

[14] Chen H, Gollnick P, Phillips RS, Eur. J. Biochem 1995, 229, 540–549. 7744078

[15] Hanks CT, Diehl ML, Craig RG, Makinen PK, Pashley DH, J. Oral Pathol. Med 1989, 18, 97–107. 2746523 10.1111/j.1600-0714.1989.tb00744.x

[16] Milic D, Demidkina TV, Faleev NG, Matkovic-Ĉalogovic D, Antson AA, J. Biol. Chem 2008, 283, 29206–29214. 18715865 10.1074/jbc.M802061200 PMC2662015

[17] Faleev NG, Ruvinov SB, Demidkina TV, Myagkikh IV, Gololobov MY, Bakhmutov VI, Belikov VM, Eur. J. Biochem 1988, 177, 395–401. 2847927 10.1111/j.1432-1033.1988.tb14388.x

[18] Faleev NG, Demidkina TV, Tsvetikova MA, Phillips RS, Yamskov IA, Eur. J. Biochem 2004, 271, 4565–4571. 15560798 10.1111/j.1432-1033.2004.04428.x

[19] Barbolina MV, Kulikova VV, Tsvetikova MA, Anufrieva NV, Revtovich SV, Phillips RS, Gollnick PD, Demidkina TV, Faleev NG, Biochimie 2018, 147, 63–69. 29183854 10.1016/j.biochi.2017.11.016

[20] Arcus VL, Prentice EJ, Hobbs JK, Mulholland AJ, Van der Kamp MW, Pudney CR, Parker EJ, Schipper LA, Biochemistry 2016, 55, 1681–1688. 26881922 10.1021/acs.biochem.5b01094

[21] Arcus VL, Pudney CR, FEBS Lett. 2015, 589, 2200–2206. 26172507 10.1016/j.febslet.2015.06.042

[22] Phillips RS, Miles EW, Holtermann G, Goody RS, Biochemistry 2005, 44, 7921–7928. 15910007 10.1021/bi050056b

[23] VonTersch RL, Secundo F, Phillips RS, Newton MG, Biomedical Frontiers of Fluorine Chemistry, American Chemical Society 1996. Ch. 7, pp. 95–104.

[24] Yamada H, Kumagai H, Kashima N, Torii H, Enei H, Okumura S, Biochem. Biophys. Res. Commun 1972, 46, 370–374. 5057881 10.1016/s0006-291x(72)80148-7

[25] Dennig A, Busto E, Kroutil W, Faber K, ACS Catal. 2015, 5, 7503–7506.

[26] Albarrán-Velo J, González-Martínez D, Gotor-Fernández V, Biocatal. Biotransform 2018, 36, 102–130.

[27] Yu Y, Zhou Q, Wang L, Liu X, Zhang W, Hu M, Dong J, Li J, Lv X, Ouyang H, Li H, Gao F, Gong W, Lu Y, Wang J Chem. Sci 2015, 6, 3881–3885. 26417427 10.1039/c5sc01126d PMC4583198

[28] Li G, Lian J, Xue H, Jiang Y, Ju S, Wu M, Lin J, Yang L, Eur. J. Org. Chem 2020, 1050–1054.

[29] Deulofeu V, Guerrero TJ, Org. Synth 1942, 22, 89.

[30] Milic D, Demidkina TV, Faleev NG, Phillips RS, Matkovic-Čalogovic D, Antson AA, J. Am. Chem. Soc 2011, 133, 16468–16476. 21899319 10.1021/ja203361g PMC3191766

[31] Vonrhein C, Flensburg C, Keller P, Sharff A, Smart O, Paciorek W, Womack T, Bricogne G Acta Crystallogr. Sect. D 2011, 67, 293–302. 21460447 10.1107/S0907444911007773 PMC3069744

[32] Kabsch W, Acta Crystallogr. Sect. D 2010, 66, 125–132. 20124692 10.1107/S0907444909047337 PMC2815665

[33] Karplus PA, Diederichs K, Science 2012, 336, 1030–1033. 22628654 10.1126/science.1218231 PMC3457925

[34] McCoy AJ, Grosse-Kunstleve RW, Adams PD, Winn MD, Storoni LC, Read RJ, J. Appl. Crystallogr 2007, 40, 658–674. 19461840 10.1107/S0021889807021206 PMC2483472

[35] Emsley P, Cowtan K, Acta Crystallogr. Sect. D 2004, 60, 2126–2132. 15572765 10.1107/S0907444904019158

[36] Afonine PV, Grosse-Kunstleve RW, Echols N, Headd JJ, Moriarty NW, Mustyakimov M, Terwilliger TC, Urzhumtsev A, Zwart PH, Adams PD, Acta Crystallogr. Sect. D 2012, 68, 352–367. 22505256 10.1107/S0907444912001308 PMC3322595

