# Supplementary material for: M379A Mutant Tyrosine Phenol-lyase from Citrobacter freundii Has Altered Conformational Dynamics
Source: Chembiochem. Author manuscript; Available in PMC 2022 Jul 8. (PMC9262865; doi:10.1002/cbic.202200028)
Supplement: Supporting information [file NIHMS1815412-supplement-Supporting_information.pdf]

Supporting information

The M379A Mutant Tyrosine Phenol-lyase from *Citrobacter freundii* Has Altered Reaction Dynamics

Robert S. Phillips[‡†], Benjamin Jones[§] and Sarah Nash[‖]

[‡]Department of Chemistry, University of Georgia, Athens, Georgia 30602 USA

[†]Department of Biochemistry and Molecular Biology, University of Georgia, Athens, Georgia 30602 USA

[‖]Department of Biology, University of Georgia, Athens, Georgia 30602 USA

[§]Department of Biological Engineering, University of Georgia, Athens, Georgia 20602 USA

Table of contents

Figure S1 3

Figure S2 4

Figure S3 5

Experimental methods 6-7

Figure S4 ^{1}H-NMR spectrum of 3-Br-DL-Phe. 8

Figure S5. ^{1}H-NMR spectrum of 3-F-L-Tyr prepared with M379A TPL. 9

Figure S6. ^{1}H-NMR spectrum of 3-Cl-L-Tyr prepared with M379A TPL. 10

Figure S7. ^{1}H-NMR spectrum of 3-methyl-L-Tyr prepared with M379A TPL. 11

Figure S8. ^{1}H-NMR spectrum of 3-MeO-L-Tyr prepared with M379A TPL. 12

Figure S9. ^{1}H-NMR spectrum of 3-MeS-L-Tyr prepared with M379A TPL. 13

References 14

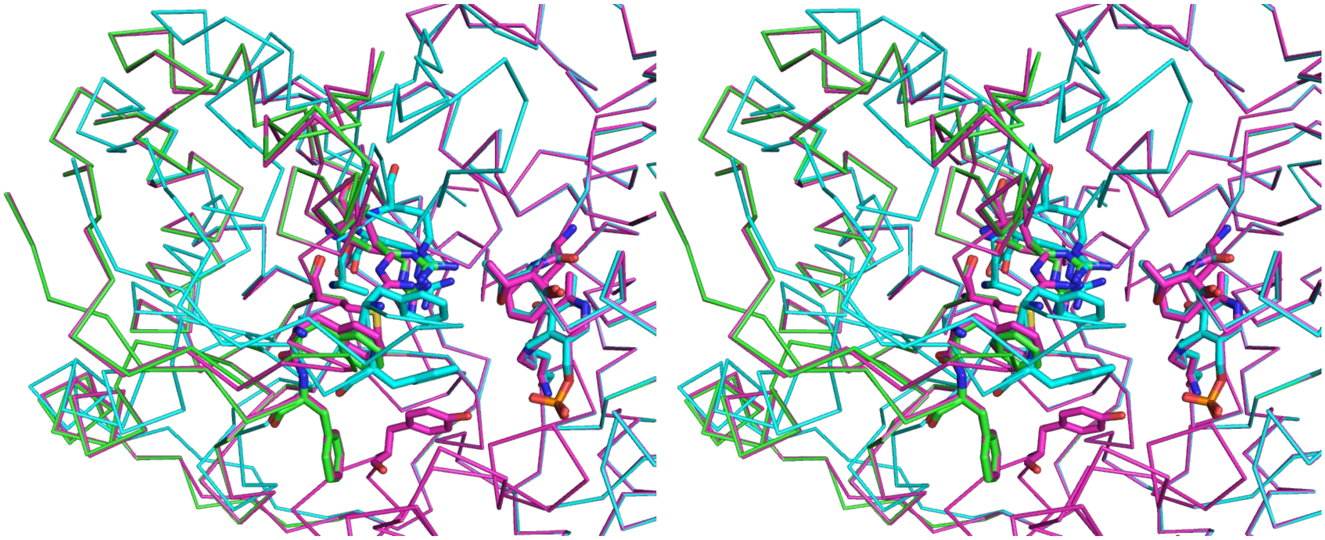

Figure S1. Crossed-eye stereo views of the overlay of the small domain of uncomplexed subunits of wild-type and M379A TPL complexes with L-methionine. Magenta, chain B of wild-type TPL; green, open conformation of chain B of M379A TPL; cyan, closed conformation of chain B of M379A TPL.

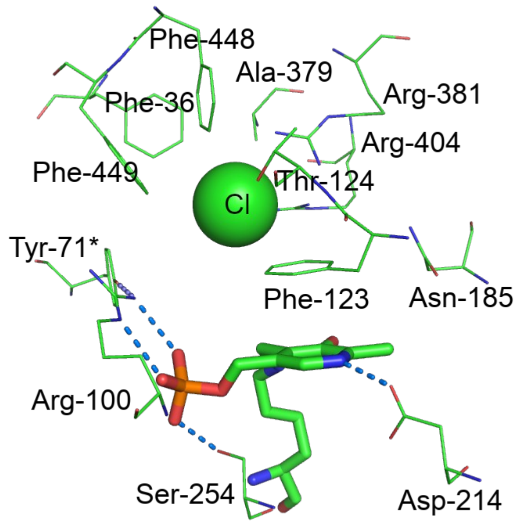

Figure S2. Crossed-eye stereo view of the structure of chain D in the M379A TPL complex with 3-bromophenylalanine. The chloride is shown as the green ball. Hydrogen bonds are indicated with blue dashes.

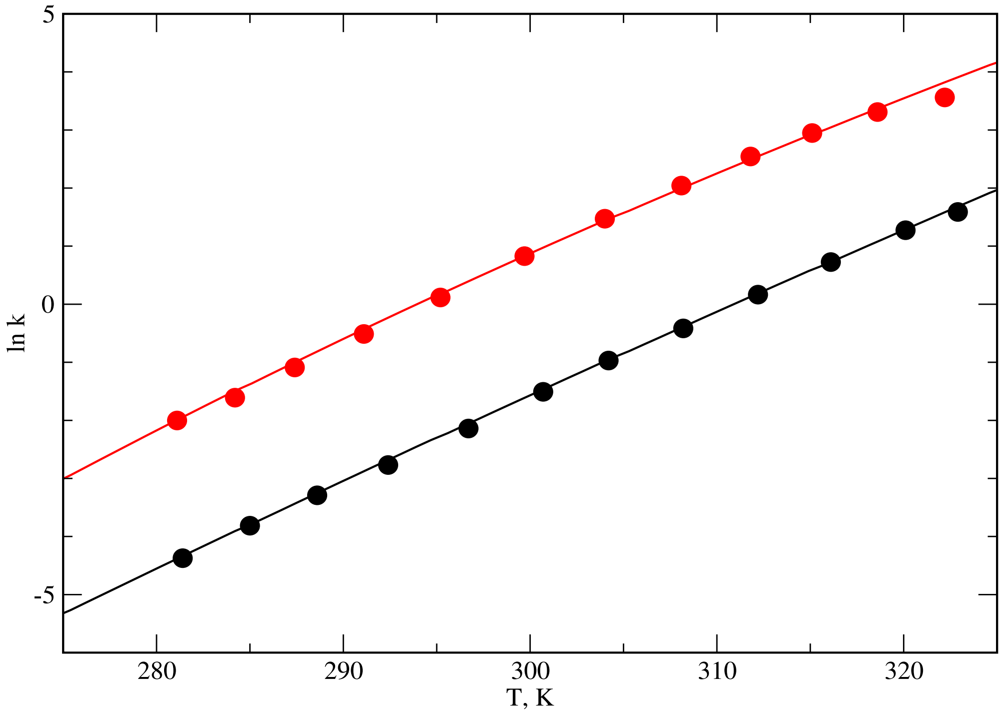

Figure S3. The effect of temperature on the formation of quinonoid intermediates with wild-type and M379A TPL. Red circles, wild type TPL; black circles, M379A TPL. The lines are the fits to the macromolecular rate equation with the parameters in the text.

Experimental Methods

Materials. Solvents, buffers and salts were obtained from commercials sources and used as obtained. 3-Fluoro, 3-chloro, 3-methyl, 3-methoxy and 3-methylthio-L-tyrosine were prepared from the corresponding phenols with M379A TPL. 3-Bromo-L-tyrosine was prepared as we have described previously [1]. L-Dopa was obtained from Sigma-Aldrich.

Preparation of M379A TPL. The mutation was performed by PCR with partially overlapping primers, as described by Naismith [2]. The primers used for mutagenesis are shown below, with the overlapping region shown in bold text. The PCR product was treated with DPN1 to remove wild-type background, and transformed into competent *E. coli* GC-5 cells. The plasmid was purified from the transformed cells and sequenced to confirm the presence of the mutation. The plasmid was transformed into *E. coli* BL21(DE3) cells for expression. The cells were grown in Studier autoinduction medium [3] at 37 C for 4 hours, followed by shaking at 26 C for 24 hours. The cells were collected by centrifugation and the mutant enzyme was purified as previously described [4].

CGT AGT GCG GAG CGC GGA ATT ATC TCT GCG GGC CGT AAT Forward

GCG CTC CGC ACT ACG TAC GCC GGT TTC CAC ATA GAT ACT Reverse

Steady-state kinetics. Steady-state kinetic data were collected at 25 ºC on a Cary 1 UV-visible spectrophotometer equipped with a 6x6 cell compartment temperature controlled by a Peltier device. The reaction mixtures for tyrosines contained 0.1 M potassium phosphate, pH 8.0, 50 µM PLP, 0.2 mM NADH, 10 µg rabbit muscle lactate dehydrogenase, 26.7 µg of M379A TPL, and various amounts of the tyrosine substrate in a volume of 600 µL. The data were fit to the Michaelis-Menten equation (Equation 1) using SciDAVis.

$$v = V_{max}*[S]/(K_m+[S]) \quad (1)$$

Stopped-flow kinetics. The reactions were performed in 0.05 M potassium phosphate, pH 8.0, in an OLIS RSM-1000 rapid-scanning stopped-flow spectrophotometer for scanning experiments, or an Applied Photophysics SX-20 instrument for single wavelength experiments. The Applied Photophysics instrument was connected to a refrigerated water bath for temperature control at 26 ºC. The rapid scanning data were collected from 240-800 nm in 256 data points at 1000 Hz, and the data were analyzed with the Global Fit

program provided by OLIS. The single wavelength kinetic data contained 1000 data points collected at 500 nm with a 4.5 nm bandpass, and were fit to one exponential process with SciDAVis [5]. The temperatures ranged between 8º and 48º C, and the enzyme was only incubated at the highest temperature for a few minutes. At least three progress curves at each temperature were fit and the rate constants were averaged.

Synthesis of 3-bromo-DL-phenylalanine. Diethyl acetamidomalonate (2.39 g, 11 mmol), 2.9 g K_2CO_3 (21 mmol) and 2.62 g (10.5 mmol) 3-bromobenzyl bromide were combined in 50 mL acetone and refluxed with stirring overnight. The heat was turned off, and the acetone was removed in vacuo. The residue was suspended in water and filtered. This crude product was dissolved in 30 mL dioxane, and 30 mL 12 M HCl was added. The cloudy mixture was then refluxed overnight. The heat was turned off, and the reaction mixture was evaporated in vacuo. The residue was suspended in 100 mL of water and extracted with 50 mL EtOAc. The pH of the aqueous layer was adjusted to ~6 (pH paper) with 30% NH_3, then it was allowed to stand overnight. The resulting precipitate was filtered, washed with a little water and EtOH, and left to dry, giving 1.61 g of white solid. The ^{1}H-NMR is consistent with the structure and is shown below.

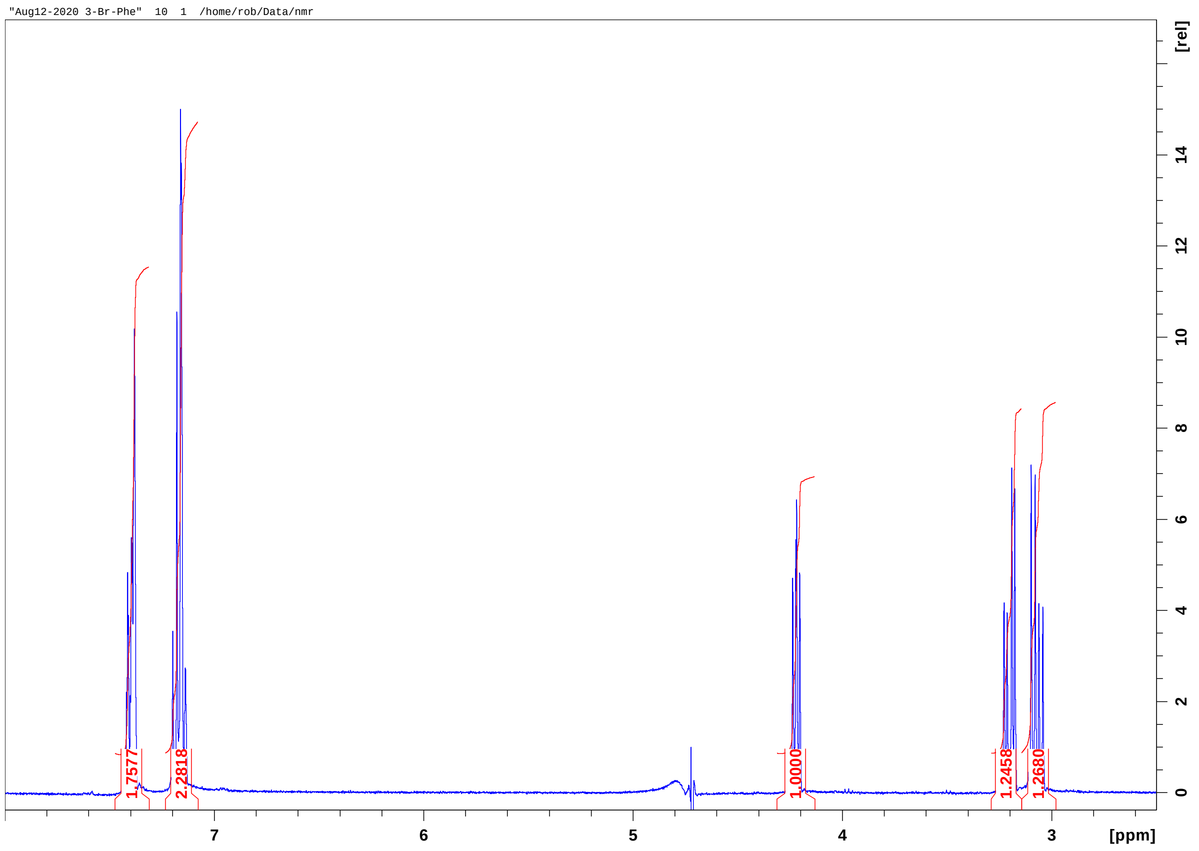

Figure S4. ^{1}H-NMR spectrum of 3-Br-DL-Phe in D_2O.

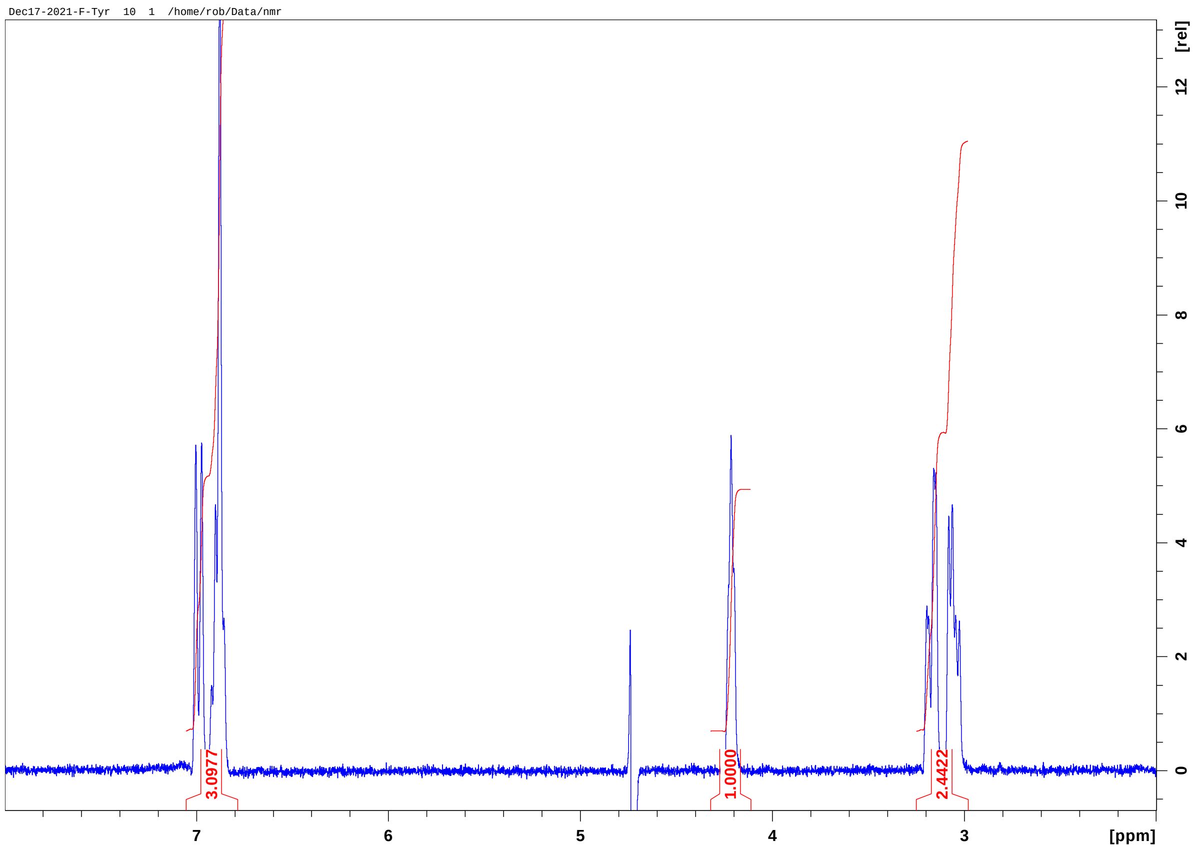

Figure S5. ^{1}H-NMR spectrum in D_2O of 3-F-L-Tyr prepared with M379A TPL.

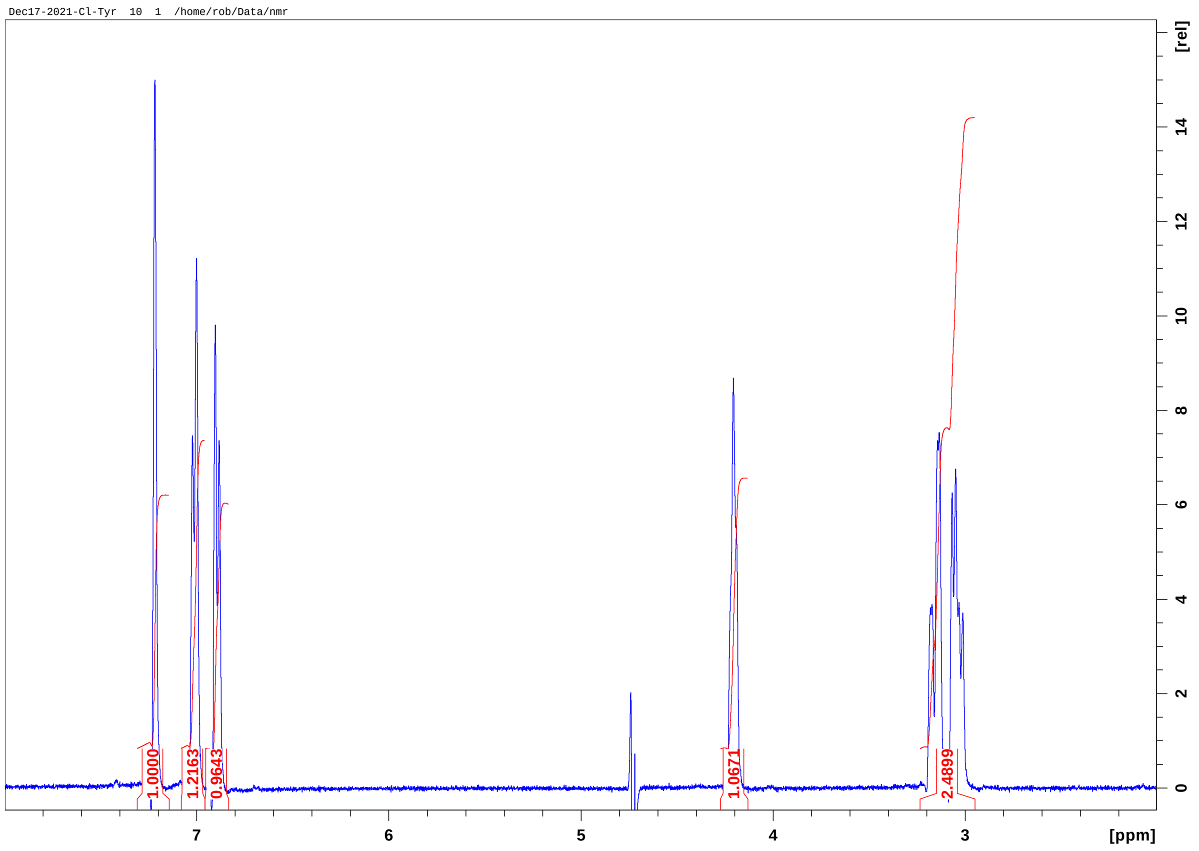

Figure S6. ^{1}H-NMR spectrum in D_2O of 3-Cl-L-Tyr prepared with M379A TPL.

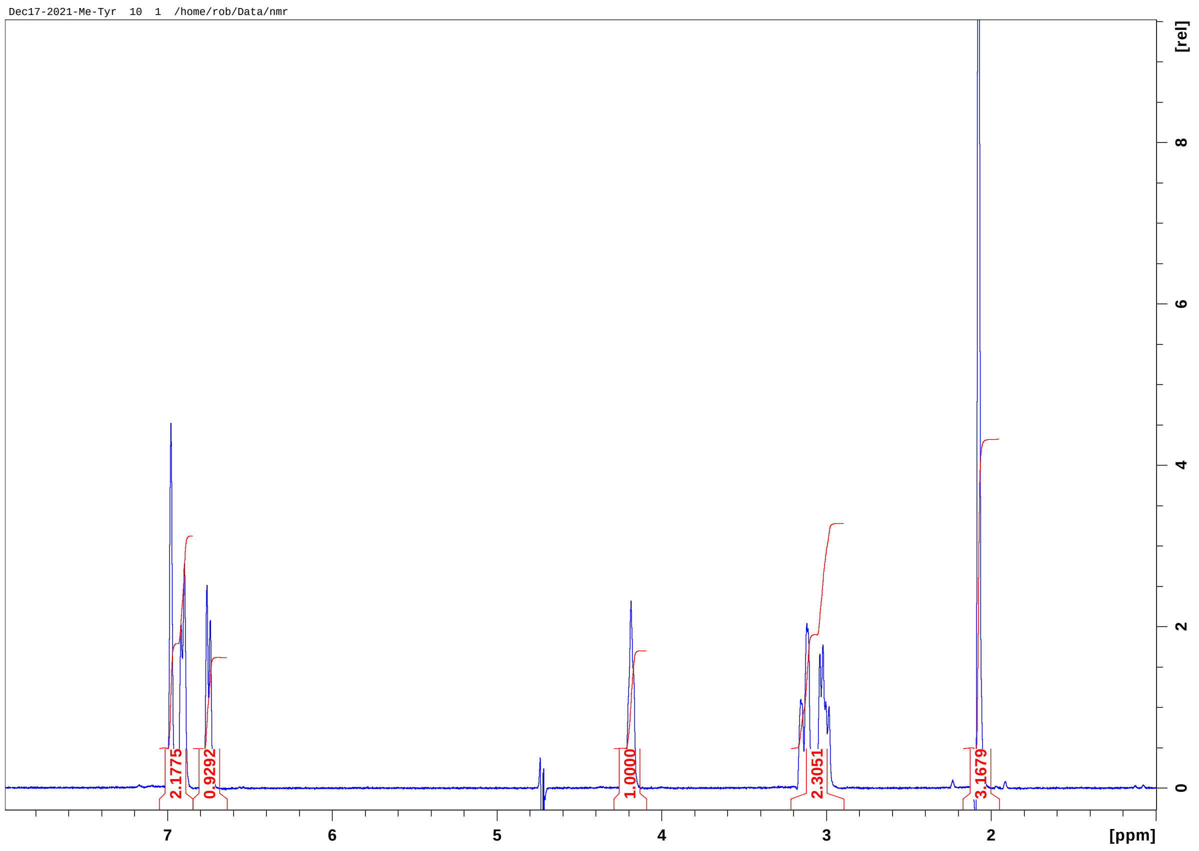

Figure S7. ^{1}H-NMR spectrum in D_2O of 3-methyl-L-Tyr prepared with M379A TPL.

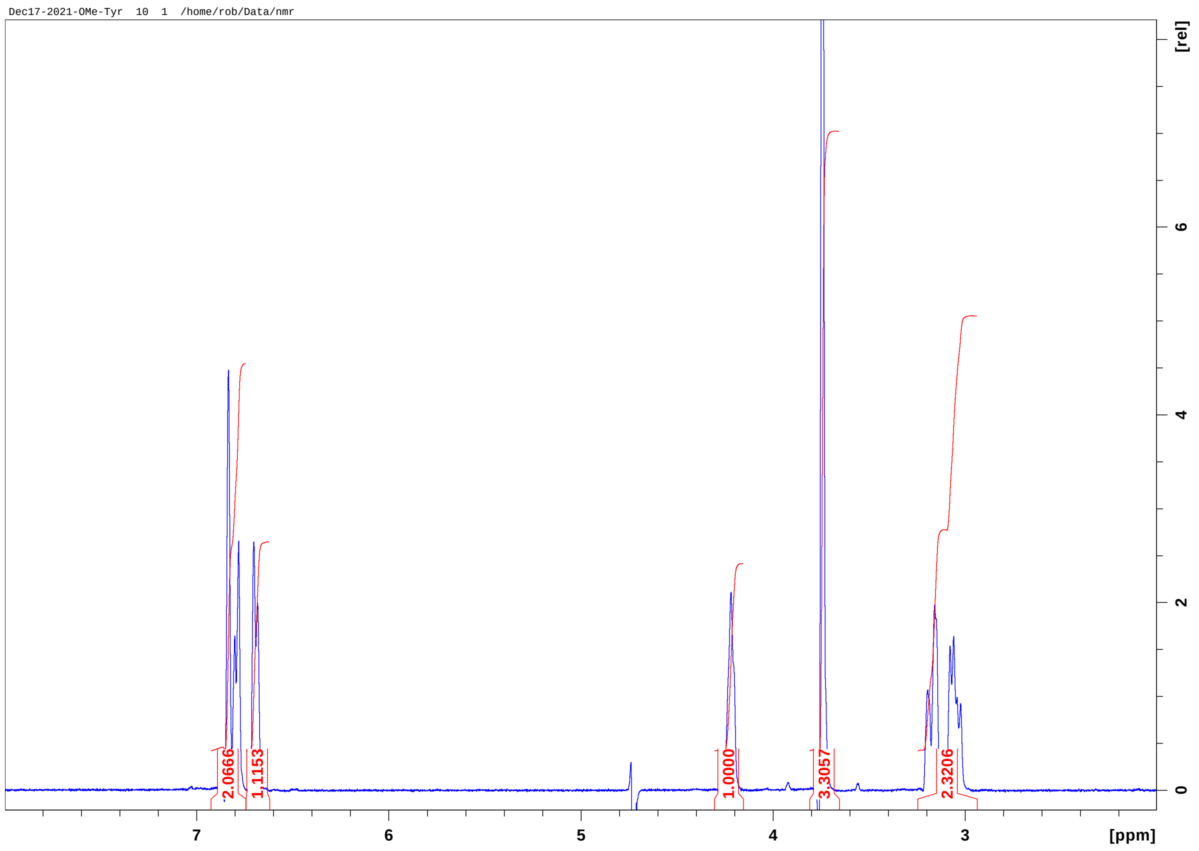

Figure S8. 1H-NMR spectrum in D_2O of 3-MeO-L-Tyr prepared with M379A TPL.

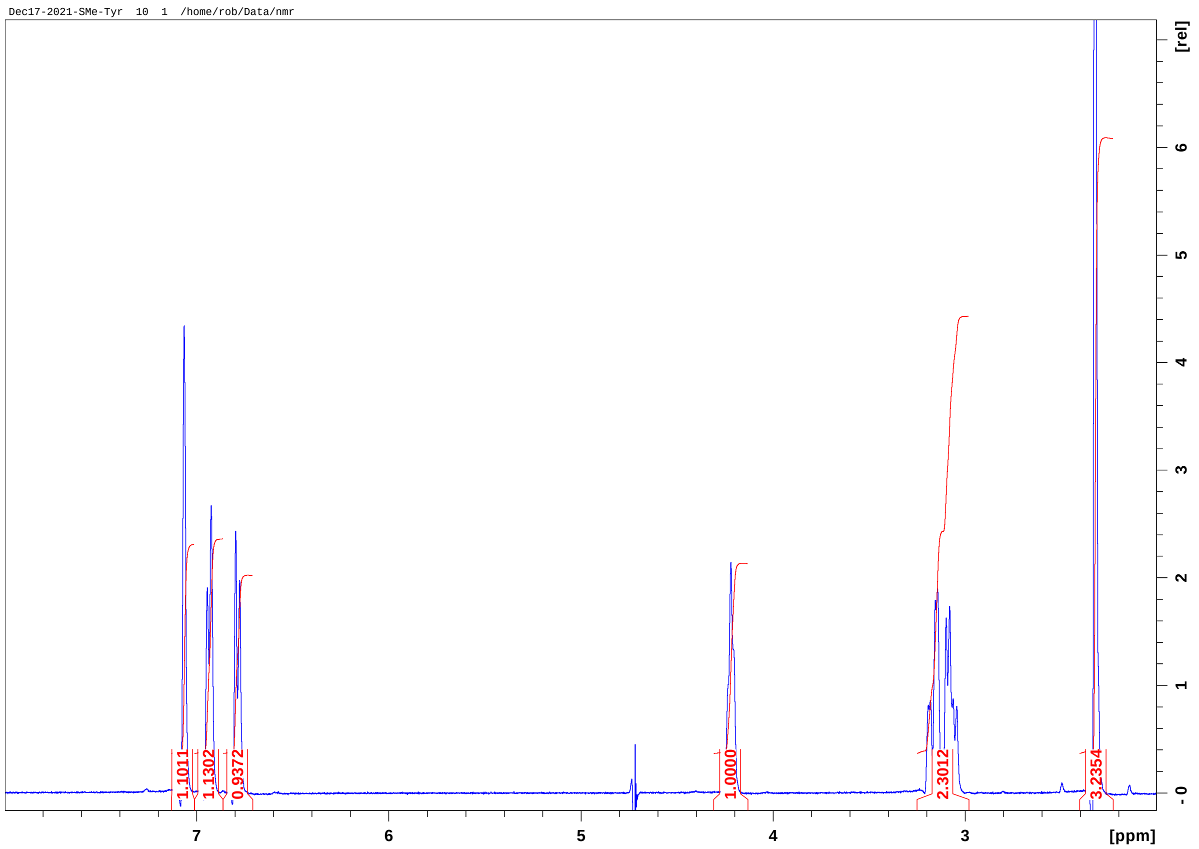

Figure S9. ^{1}H-NMR spectrum in D_2O of 3-MeS-L-Tyr prepared with M379A TPL.

References

[1] R. S. Phillips, S. Busby, L. Edenfield, K. Wickware, *Amino Acids* **2013,** *44*, 529-532.

[2] H. Liu, J. H. Naismith, *BMC Biotechnology* **2008**, *8*, 1-10.

[3] F. W. Studier, *Prot. Exp. Purif*. **2005**, *41*, 207-234.

[4] H. Chen, P. Gollnick, R. S. Phillips, *Eur. J Biochem*. **1995**, *229*, 540-549.

[5] http://scidavis.sourceforge.net/index.html
